# Does kidney biopsy in pediatric lupus patients “complement” the management and outcomes of silent lupus nephritis? Lessons learned from a pediatric cohort

**DOI:** 10.1007/s00467-022-05859-w

**Published:** 2023-01-23

**Authors:** Sai Sudha Mannemuddhu, Lawrence R. Shoemaker, Shahab Bozorgmehri, R. Ezequiel Borgia, Nirupama Gupta, William L. Clapp, Xu Zeng, Renee F. Modica

**Affiliations:** 1 Department of Pediatrics, Division of Nephrology, University of Florida-School of Medicine, Gainesville, FL USA; 2 Present Address: Pediatric Nephrology, East Tennessee Children’s Hospital, 2100 Clinch Avenue, MOB, Suite 310, Knoxville, TN 37916 USA; 3 Department of Medicine, University of Tennessee, Knoxville, TN USA; 4 Department of Medicine, Division of Nephrology, University of Florida-School of Medicine, Gainesville, FL USA; 5 Department of Pediatrics, Division of Rheumatology, University of Florida-School of Medicine, Gainesville, FL USA; 6 Present Address: Department of Pediatrics, Division of Allergy, Immunology, and Rheumatology, University Hospitals Rainbow Babies and Children’s Hospital, Cleveland, OH USA; 7 Present Address: Blue Jay Pediatrics, Leesburg, VA USA; 8 Department of Pathology, Immunology, and Laboratory Medicine, University of Florida-School of Medicine, Gainesville, FL USA

**Keywords:** Silent lupus nephritis (SLN), Overt lupus nephritis (OLN), Low complement, Kidney biopsy, Pediatric systemic lupus erythematosus (pSLE)

## Abstract

**Background:**

Silent lupus nephritis (SLN) is systemic lupus erythematosus (SLE) without clinical and laboratory features of kidney involvement but with biopsy-proven nephritis. This study aims to describe and compare the baseline characteristics and outcomes of pediatric SLN with overt LN (OLN) and to identify associated risk factors and biochemical markers.

**Methods:**

In this retrospective, observational study, multivariate logistic regression and receiver operating characteristic (ROC) analyses studied age, sex, race, serum complements, anti-double-stranded-DNA antibody, anti-Smith antibody, eGFR, and proliferative nephritis.

**Results:**

In our cohort of 69 patients, 47 were OLN, and 22 were SLN. OLN (*OR* = 4.9, *p* = 0.03) and non-African Americans (AA) (*OR* = 13.0, *p* < 0.01) had higher odds, and increasing C3 and C4 were associated with lower odds of proliferative nephritis (*OR* 0.95 and 0.65 per one unit increase in C3 and C4, respectively, *p* < 0.01). They demonstrated a good discriminative ability to detect proliferative nephritis as assessed by the area under the ROC curve (C3 = 0.78, C4 = 0.78). C3 and C4 in proliferative SLN and OLN were comparable and significantly lower than their non-proliferative counterparts. No association was observed between age, sex, anti-double-stranded-DNA antibody, anti-Smith antibody, eGFR, and proliferative nephritis. Proliferative SLN and OLN patients received similar treatments. Adverse events were identified in the proliferative OLN only.

**Conclusions:**

Lower complement levels are associated with proliferative lesions in pediatric LN—both SLN and OLN. The non-AA population had higher odds of having proliferative nephritis than the AA. Prospective, randomized, long-term follow-up of proliferative SLN patients is needed to ascertain the beneficial effect of early diagnosis and treatment.

**Graphical abstract:**

**Figure:**
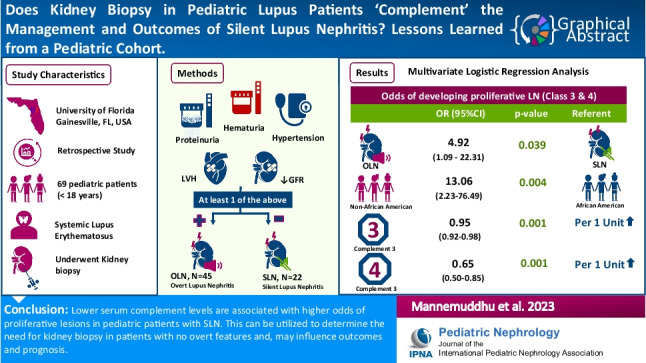
A higher resolution version of the Graphical abstract is available as Supplementary information

**Supplementary Information:**

The online version contains supplementary material available at 10.1007/s00467-022-05859-w.

## Introduction

Systemic lupus erythematosus (SLE) is a chronic autoimmune disease with multisystem involvement that often injures the kidney. Pediatric SLE (pSLE) accounts for 15–20% of all cases of SLE [1–5]. pSLE is a rare disease with an incidence of 0.3–0.9 per 100,000 children years and a prevalence of 3.3–8.8 per 100,000 children. pSLE is abrupt in onset and severe compared with adult lupus [2, 4–6].

Lupus nephritis (LN) is an important manifestation of lupus and a predictor of poor disease prognosis. The prevalence of LN is higher in pSLE compared to adult SLE [2] and is reported as high as 20–75% with significant ethnic variations [4, 6–8]. More than 90% of those destined to develop kidney disease will do so within the first 2 years after SLE diagnosis [9]. About 10–44% of pSLE patients will progress to kidney failure depending on the disease severity and management [5, 10]. Kidney biopsy is the gold standard for diagnosing LN [11].

LN can present with or without overt features, such as abnormal urinary sediment, impaired kidney function, including proteinuria/albuminuria, microscopic (or rarely macroscopic) hematuria, hypertension (HTN), and/or elevated serum creatinine. When LN is presented with any of the above features, it is called overt lupus nephritis (OLN); in the absence, it is called silent lupus nephritis (SLN). This concept of SLN, apparent only upon kidney biopsy, has been recognized since 1976, with a few published studies mainly in the adult population [12–14]. To identify pSLE patients with nephritis, some centers have instituted baseline kidney biopsy at SLE diagnosis in children with the significant clinical and serologic activity of SLE, regardless of the presence or absence of overt kidney involvement [12]. These baseline kidney biopsies have led to the recognition of patients with SLN; however, with prospective randomized studies, it would be easier to determine which patients would benefit from such biopsies. The prevalence of SLN is unknown; however, its actual prevalence is thought to be higher than reported [13] since invasive kidney biopsy is the only way to diagnose SLN at this time.

Early identification and treatment of patients with LN correlate with early remission. Remission at 24 months after diagnosis is associated with a better prognosis, as shown in some adult studies [15]. Undertreated LN is associated with mortality directly attributable to kidney disease and is seen in about 5–25% of patients within 5 years of the onset of the disease [16, 17].

This study aims to retrospectively describe and compare the clinical and laboratory characteristics of SLN and OLN and to identify risk factors and biochemical markers of proliferative LN in our pSLE cohort, which can aid in early diagnosis and management.

## Methods

After the institutional review board’s (IRB201802407) approval, patients with pSLE (≥ 1 year old and ≤ 18 years old) [18] were identified retrospectively from the electronic medical health record system located at the University of Florida from 2011 to 2018 by using diagnostic (ICD 9 and ICD 10) and CPT codes for SLE, kidney biopsy, and nephritis (Supplementary Table S1). The study was conducted according to the principles of the Declaration of Helsinki.

### Inclusion criteria

Patients with lupus nephritis and kidney biopsies were included who had a previous diagnosis of SLE based on the American College of Rheumatology (ACR) 1997 or Systemic Lupus International Collaborating Clinics (SLICC) 2012 classification criteria [19, 20].

### Exclusion criteria

Pregnant patients, patients diagnosed with SLE but who did not undergo kidney biopsy or whose biopsy report is not available, patients diagnosed with SLE after 18 years of age, patients with drug-induced SLE, and patients with mixed connective tissue disease (MCTD) overlap syndromes were excluded. MCTD is a syndrome with overlapping features of SLE, systemic sclerosis, polymyositis, and antibodies to RNase-sensitive extractable nuclear antigen [21]. Overlap syndrome is the occurrence of at least two connective tissue diseases at the same time or at different times in one patient [22].

### Data collection

Demographics, clinical manifestations, laboratory results, and kidney biopsy findings were collected from the charts of the patients qualified for this study. For patients who underwent kidney biopsy, abnormal urinalysis and impairment of kidney function were defined based on the following criteria upon consensus/agreement of the investigators/having at least 1 of the following four criteria qualified as OLN: (i) urine protein creatinine ratio (UPCR) > 0.2 mg/mg [23]; (ii) active urinary sediments (RBC ≥ 5 per HPF, WBC ≥ 5 per HPF, dysmorphic RBCs (acanthocytes), isomorphic RBCs, presence of casts, etc.) [24]; (iii) diagnosis of HTN or presence of left ventricular hypertrophy (LVH) in the echocardiogram. Charts were reviewed for the presence of the CPT codes for the diagnosis of HTN or LVH (Supplementary Table S1). In the absence of these diagnoses, the mean of multiple measurements (manual and automated) that were available for review were used. If the mean BP was > 95th percentile for the age, height, and gender (< 13 years old) or a single point measure for ≥ 13 years old, they were classified as HTN (AAP 2017 HTN guidelines [25]). Also, echocardiogram reports were reviewed for the presence of LVH. (iv) A glomerular filtration rate (GFR) < 90 ml/min/1.73 m^2^. Depending on the availability, 24-h urine creatinine clearance or bedside Schwartz formula was used to calculate GFR.

Two pathologists reviewed all the reports to avoid bias and re-classified LN based on the new International Society of Nephrology/Renal Pathology Society (ISN/RPS) classification for lupus nephritis 2016. Both pathologists were blinded to the clinical history of the patients.

### Data analysis

Descriptive statistics were tabulated, and values were reported as medians (interquartile ranges) or numbers (%). The multivariate logistic regression analysis evaluated the association between demographics, clinical characteristics, laboratory features, and pathology findings (proliferative nephritis). Values are expressed as odds ratio (OR) and 95% confidence interval (CI). Due to multicollinearity, analysis was conducted with C3 and C4 separately. Receiver operating characteristic (ROC) analysis was used to assess the predictive performance of C3 and C4 to detect proliferative nephritis at biopsy. Results were represented as the areas under the curve (AUC). A *p*-value < 0.05 was considered statistically significant. Statistical analyses were performed using Statistical Analysis Software (SAS) version 9.4 (SAS Institute Inc, Cary, NC, USA).

## Results

**Fig. 1:**
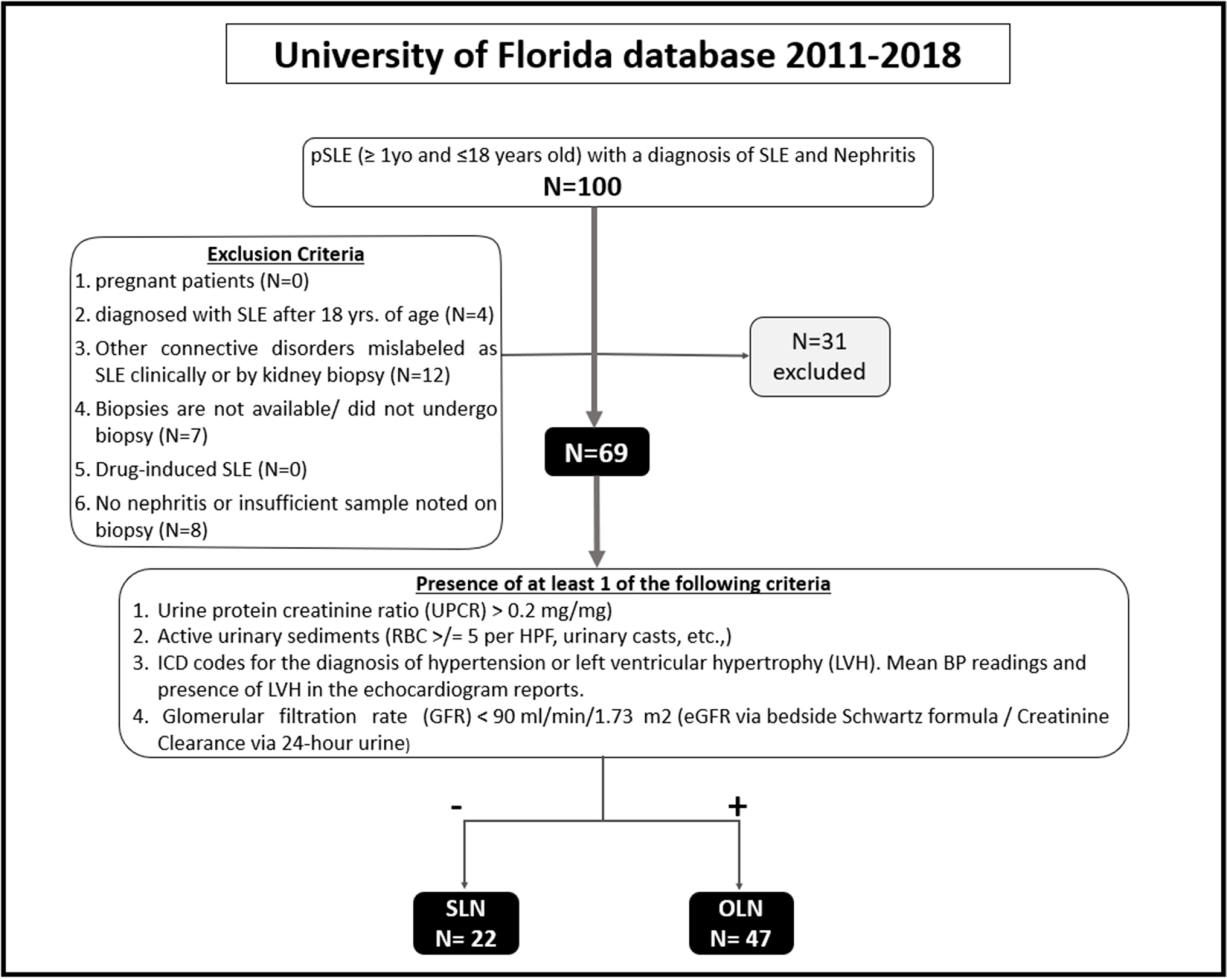
Inclusion and exclusion criteria followed by the final number of eligible patients enrolled in the study. SLN, silent lupus nephritis; OLN, overt lupus nephritis

One hundred patients from the University of Florida database were identified between 2011 and 2018. Sixty-nine patients met the inclusion criteria and were found to have histopathologic evidence of LN (Fig. 1). Of these, 22 (32%) had SLN, and 47 (68%) had OLN. Class II LN was the predominant type in SLN patients (*n* = 11, 50%), whereas class IV was predominant in OLN patients (*n* = 19, 40%). In the SLN group, the frequencies of ISN/RPS class I to V nephritis were as follows: 3 (14%), 11 (50%), 4 (18%), 2 (9%), and 2 (9%), respectively (Table 1). Either proliferative or membranous accounted for 36% (*n* = 8) of SLN, and proliferative nephritis represented approximately a quarter (27%, *n* = 6) of the SLN group in our cohort. In summary, 1 out of every 2.7 patients without any biochemical or clinical findings of kidney involvement who underwent kidney biopsy was found to have proliferative or membranous SLN.

**Table 1:** Demographics, serological, clinical, and histological findings in the pSLE LN cohort at the University of Florida

Silent vs. overt lupus nephritis				
Factor		Silent lupus nephritis	Overt lupus nephritis	*P*-value
*N*		22	47	
Female		18 (82%)	40 (85%)	0.737
Race	Asian	0	1 (2%)	0.307
	African-American	13 (59.5%)	25 (53%)	
	Hispanic	4 (18%)	9 (19%)	
	Other	4 (18%)	3 (7%)	
	Caucasian	1 (4.5%)	9 (19%)	
Age, mean (*SD*)	At diagnosis	12.6 (4.5)	13.5 (3.2)	0.358
	At biopsy	13 (4.6)	14 (3.4)	0.219
Complement (mg/dL), median (IQR)	C3	59.5 (34–77)	48 (37–74)	0.984
	C4	6.5 (3–8)	7 (4–10)	0.403
dsDNA positive		18 (82%)	36 (77%)	0.759
Anti-Smith Ab		17 (77%)	33 (70%)	0.628
Anti-RNP Ab		20 (91%)	33 (70%)	0.05
Anti-SSA Ab		11 (50%)	24 (51%)	0.8
Anti-SSB Ab		15 (68%)	29 (62%)	0.4
Anti-phospholipid Ab		15 (68%)	37 (79%)	0.7
eGFR (mL/min/1.73m^2^), median (IQR) at biopsy		126 (116–138)	111.5 (84–136)	0.041
Hypertension		0	9 (19%)	0.0489
Random UPCR mg/mg, median (IQR)		0.12(0.07–0.17)	1.2(0.3–3.3)	< 0.001
Biopsy results	Class I	3 (14%)	1 (2%)	
	Class II	11 (50%)	10 (21%)	
	Class III	4 (18%)	8 (18%)	
	Class IV	2 (9%)	19 (40%)	
	Class V	2 (9%)	9 (19%)	
	Class VI	0	0	

Demographics, antibody panel, eGFR, hypertension, urinary findings, and histopathology findings in SLN vs. OLN patients. OLN was seen in 68% of patients, and SLN in 32%. eGFR is normalized to BSA

*eGFR*, estimated glomerular filtration rate; *IQR*, interquartile ranges; *UPCR*, urine protein creatinine ratio

In the OLN group, the frequencies of ISN/RPS class I to V nephritis were as follows: 1 (2%), 10 (21%), 8 (18%), 19 (40%), and 9 (19%), respectively (Table 1). Proliferative, membranous, or mixed LN was noted in 77% (*n* = 36) of OLN patients. There were five patients with mixed class in the OLN cohort, three patients with class IV and V, one with class III and V, and one with class II and V. Patients with class II and V were grouped under class V; those with class III and V lesions were grouped under class III; and those with class IV and V lesions were grouped under class IV. No class VI patients were identified via kidney biopsy. The NIH median activity index of the proliferative SLN group was 5.5 (IQR 4–9), and that of proliferative OLN was 8 (IQR 5–11), which was not statistically significant (*p* = 0.37). The NIH median chronicity index was 0 in proliferative SLN and 1 in proliferative OLN groups (*p* = 0.05) (Table S2). All the pSLE patients in this study who underwent kidney biopsy were new onset, biopsied at presentation (75.5%), or within 1 year of presentation (10%). The remainder (14.5%) were biopsied between 1 and 6 years of presentation.

**Fig. 2:**
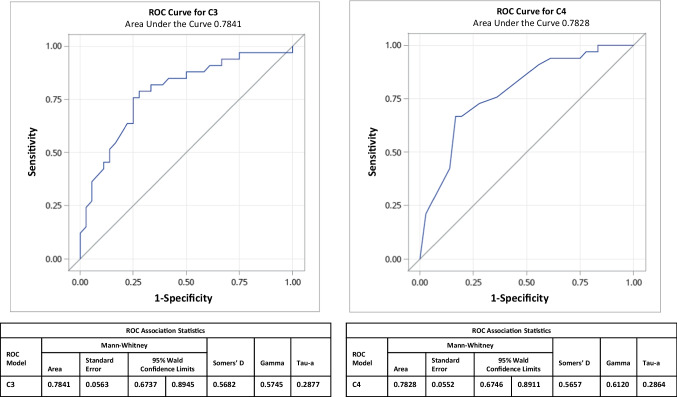
Predictive performance of C3 and C4 at biopsy to detect proliferative nephritis using ROC curve analysis

Multivariate logistic regression analysis (Tables 2 and 3) was performed to identify the factors associated with proliferative nephritis. In this cohort of patients who underwent kidney biopsy, patients with OLN had higher odds (*OR* = 4.92, *p* = 0.039) of having proliferative nephritis than SLN. The non-African American (non-AA) population had higher odds (*OR* = 13.06, *p* = 0.004) of having proliferative nephritis than the AA population, though the AA population accounted for > 50% of the patients in our cohort. There is no association between age, gender, presence of anti-ds-DNA antibody (Ab) or anti-Sm Ab, a decline in eGFR, and the odds of proliferative nephritis. Increasing levels of C3 and C4 are associated with lower odds of proliferative nephritis (OR 0.95 per one unit increase in C3, *p* < 0.01; *OR* 0.65 per one unit increase in C4, *p* < 0.01). Upon ROC analysis, C3 and C4 showed a good discriminative ability to detect proliferative nephritis, as demonstrated by AUC (C3 = 0.7841, C4 = 0.7828) (Fig. 2). Also, the median C3 and C4 levels of both proliferative OLN and SLN were similar (median C3 for OLN was 44 (27–54) and for SLN was 32.5 (24–34); median C4 for OLN was 2.5 (2–4), and SLN was 4 (3–8)) and were significantly lower (*p* < 0.05) than their non-proliferative counterparts (median C3 for OLN was 72 (52–88), and SLN was 63 (46.5–88.5); median C4 for OLN was 8 (4–9.5), and SLN was 9.5 (7–16.5)) (Tables S3 and S4).

**Table 2:** Predictors of proliferative nephritis on multivariable logistic regression analysis with C3^*^

	Referent	Odds ratio (95% CI)	*P*-value
Overt LN	Silent LN	4.92 (1.09–22.31)	**0.039**
Age	per one year	1.00 (0.82–1.24)	0.975
Female	Male	5.93 (0.57–61.74)	0.137
Not African-American	African-American	13.06 (2.23–76.49)	**0.004**
dsDNA Ab, positive	Negative	1.08 (0.20–5.86)	0.927
Anti-Smith Ab, positive	Negative	0.29 (0.05–1.67)	0.165
C3	Per one unit	0.95 (0.92–0.98)	**0.001**
eGFR	Per one unit	0.99 (0.97–1.01)	0.294

Multivariate logistic regression analysis demonstrating the association between baseline demographics, laboratory features, pathology findings, and proliferative nephritis. Values in bold indicate significant association

^*^C3 is included in the model, but C4 is not included due to multicollinearity

**Table 3:** Predictors of proliferative nephritis on multivariable logistic regression analysis with C4^*^

	Referent	Odds ratio (95%CI)	*P*-value
Overt LN	Silent LN	10.23 (1.90–55.10)	**0.007**
Age	per one year	0.93 (0.75–1.14)	0.464
Female	Male	17.62 (1.35–229.61)	0.029
Not African-American	African-American	12.14 (1.75–84.43)	**0.012**
dsDNA Ab, positive	Negative	0.59 (0.09–3.82)	0.579
Anti-Smith Ab, positive	Negative	0.25 (0.03–1.91)	0.183
C4	Per one unit	0.65 (0.50–0.85)	**0.001**
eGFR	Per one unit	0.98 (0.96–1.01)	0.145

Multivariate logistic regression analysis demonstrating the association between baseline demographics, laboratory features, pathology findings, and proliferative nephritis. Values in bold indicate significant association

^*^C4 is included in the model, but C3 is not included due to multicollinearity

SLN and OLN groups did not show any significant difference in the frequency of clinical features such as malar rash, fevers, headaches, mucosal ulcers, arthritis, serositis, or myositis (Table S5). Similarly, there was no statistically significant difference between the percentage of patients with anemia, leucopenia, and thrombocytopenia between the SLN and OLN groups.

Similarly, when proliferative SLN and OLN were compared histopathologically, the median activity and chronicity indices were higher in proliferative OLN compared to proliferative SLN. The rest of the clinical, hematological, and serological findings were similar in both groups (Table S6).

## Outcomes

After 1 year of follow-up, we compared the treatment received, lab results, eGFR, presence of HTN, urinary sediment, and adverse events in patients with proliferative (class III and IV) and class V, SLN, and OLN. One out of 8 patients with proliferative SLN and 3 out of 36 proliferative OLN patients were lost to follow-up. The percentage of patients who received pulse doses of methylprednisolone, mycophenolate mofetil (MMF), rituximab, and cyclophosphamide were similar between the SLN and the OLN groups (proliferative and membranous) (Table 4). One patient in the SLN group and two in the OLN group received other biologic agents. None of the proliferative SLN patients received plasmapheresis (PLEX) or other disease-modifying anti-rheumatic drugs (DMARDs), while 3% and 6%, respectively, of the proliferative OLN group, received them. Among mixed class patients, mixed class III and IV were treated as proliferative nephritis. Patients with mixed class II and V lesions were treated as class V. Their outcomes were assessed as their respective class III, IV, or V.

**Table 4:** Outcomes of patients with proliferative silent and overt lupus nephritis after 1 year of follow-up

			Silent LN (class III, IV, and V)	Overt LN (class III, IV, and V)
*N*			7	33
Treatment (induction and maintenance)	Pulse methylprednisolone		7 (88%)	32 (89%)
	Mycophenolate mofetil		7 (88%)	31 (86%)
	Rituximab		5 (63%)	22 (61%)
	Cyclophosphamide		5 (63%)	22 (61%)
	Other biologics		1 (13%)	2 (6%)
	Plasmapheresis		0	1 (3%)
	Other DMARDs (methotrexate, tacrolimus, azathioprine)		0	2 (6%)
eGFR (mL/min) normalized to BSA	CKD stage 1	Hyperfiltration	1 (13%)	7 (19%)
		eGFR 90–120	6 (75%)	16 (44%)
	CKD stage 2		0	7 (19%)
	CKD stage 3		0	1 (3%)
	CKD stage 4		0	0
	CKD stage 5/kidney failure		0	2 (6%)
Hypertension			0	2 (6%)
LVH			0	2 (6%)
Hematuria			0	15 (42%)
UPCR mg/mg Median (IQR)			0.08 (0.05–0.14)	1.15 (0.4–3.7)
C3 mg/dL Median (IQR)			124 (86–151)	124.5 (88–149)
C4 mg/dL Median (IQR)			24 (12–26)	27.5 (16–34)
dsDNA positive			2 (29%)	14 (39%)
Adverse events 1-year post-biopsy and post-treatment	Hypogammaglobulinemia		0	11 (31%)
	Thrombosis		0	5 (14%)
	Sepsis		0	2 (6%)
	Herpes zoster		0	1 (3%)
	Mortality		0	1 (3%)
	Diabetes mellitus		0	1 (23%)
	PRES		0	1 (3%)

Treatment, laboratory findings, clinical findings, and adverse events in proliferative SLN and OLN patients during 1 year of follow-up

*DMARDs*, disease-modifying anti-rheumatological agents; *CKD*, chronic kidney disease; *LVH*, left ventricular hypertrophy; *eGFR*, estimated glomerular filtration rate; *IQR*, interquartile ranges; *UPCR*, urine protein creatinine ratio; *PRES*, posterior reversible encephalopathy syndrome; *APL*, anti-phospholipid antibody syndrome

After a year of follow-up, all patients in the proliferative SLN group had eGFR ≥ 90 mL/min (CKD stage 1), whereas OLN patients showed eGFR ranging from CKD stage 1 to 5 (stage 1: 21%, stage 2: 49%, stage 3: 3%, stage 4: 6%, stage 5/kidney failure: 6%). None of the patients with SLN had HTN during a 1 year follow-up (similar to before treatment), whereas only 6% of patients with OLN had HTN (compared to 26% before treatment). Hematuria persisted in 46% of patients with OLN.

After 1 year of treatment, median C3 and C4 levels normalized and were similar between proliferative SLN and OLN, with median C3 being 124 and 124.5 mg/dL and median C4 levels being 24 and 27.5 mg/dL, respectively.

No adverse events were noted in the SLN group. In the OLN group, up to 31% of patients developed several adverse events: hypogammaglobulinemia in 31%, deep venous thrombi in 14%, development of anti-phospholipid antibody syndrome in 12%, sepsis in 6%, diabetes mellitus in 3%, posterior reversible encephalopathy syndrome (PRES) in 3%, and mortality was seen in 3%.

## Discussion

In this study, we identified 1 out of every 2.7 patients without any biochemical or clinical findings of kidney involvement who underwent kidney biopsy and were found to have proliferative or membranous SLN, which is very significant. Either proliferative or membranous LN was noted in 1 out of every 1.9 OLN patients, which was predictable owing to the aggressive presentation of this group.

Currently, kidney biopsy is the gold standard for diagnosing lupus nephritis, and formal guidelines for kidney biopsy in the absence of overt kidney involvement do not exist; the frequency of performing kidney biopsy may vary among institutions. The SLN patients in this study were referred for a biopsy to establish a firm diagnosis of SLE in patients when overlap, mixed, or other connective tissue disorders were being considered in the differential diagnosis and were the physician’s preference in some cases. Patients with histopathologic evidence of SLN (22, 32%) in our study were lower when compared to 55% of SLN, seen in an adult study from Japan, but higher than other pediatric and adult studies where proliferative SLN accounted for 12.5–24% of the SLN population [12, 13, 26].

In light of this, we endeavored to uncover biomarkers to differentiate proliferative from non-proliferative SLN patients since the former could benefit from intensified treatment as multiple studies (predominantly from the adult population) have shown that decreased levels of complement components C1q, C3, C4, and CH50 tend to correlate with disease activity in patients with active OLN, [13, 27–29]. Still, the data regarding SLN is limited [12, 13]. We observed that none of the serologic auto-antibody markers in our study could accomplish this in light of prior reports suggesting an association of high levels of ds-DNA [30] and anti-Sm antibodies with SLN [13]. Upon multivariate logistic regression, only lower C3 and C4 levels were associated with higher odds of proliferative nephritis. Median C3 and C4 of proliferative SLN and OLN were comparable and significantly lower than their non-proliferative counterparts. Several authors have reported the association of severe nephritis with low C3 [12, 13, 30–32]; however, disagreement persists over a similar association with low C4 [12, 32]. Low C3 represents immune consumption and deposition in the kidneys; however, a primary complement deficiency seen in a subset of pSLE patients can be a confounding factor, particularly with low C4 levels [33]. Demographic characteristics in our study showed that the non-AA population had higher odds of having proliferative LN than the AA population, unlike prior studies [6].

It is unknown how long patients with SLN would have remained clinically dormant in the negative laboratory and clinical findings had the biopsy not been performed, given that predominant nephritis was class II in the SLN group, similar to some adult studies [34]. The finding that the prevalence of proliferative nephritis in the OLN group was more than double the prevalence in the SLN group and that the nephritis was more severe in the OLN group as determined by activity index was expected (median activity index 8 vs. 5.5). The median chronicity index was 0 for proliferative SLN and 1 for proliferative OLN (Table 1) which is plausible since most (85.5%) of the patients with LN included in our cohort were biopsied within 1 year of diagnosis of SLE.

Both the proliferative SLN and OLN groups received similar treatment and hypocomplementemia resolved after a year of treatment (Table 4). Our center utilized a multi-targeted protocol for induction and maintenance, showing better outcomes and safety profiles than in some pediatric studies in children with severe proliferative LN [35, 36]. All proliferative SLN patients remained in stage 1 CKD, but only 21% of OLNs were in stage 1. Treatment decreased HTN prevalence from 26 to 6% in the proliferative OLN group (Table S2 and Table 4). No adverse events were noted in the proliferative SLN group; however, the proliferative OLN group had various adverse events, including mortality.

Patients with proliferative SLN have excellent 1-year outcomes. It is unclear if this is due to early kidney biopsy and treatment or if the outcomes of proliferative SLN may be better anyway when compared to proliferative OLN, and this proliferative SLN cohort may be overtreated. A retrospective study of 20 adult SLN patients (3 proliferative SLN) with 10-year follow-up showed 0% mortality or kidney failure [37], similar to ours. Another retrospective study of adult SLN patients (*n* = 20 patients and 1 case of proliferative nephritis) reported that after a median follow-up of 13 years, two patients were lost to follow-up, three patients died of non-kidney-related causes, and the remaining 15 had normal kidney function and urinalysis. They concluded that SLN plays a minor clinical role in the outcomes of these patients, although the majority of SLN patients in this cohort had non-proliferative LN [38]. The same study also conducted a literature review and found that an additional 193 patients (30% were proliferative SLN) had kidney and patient survival rates of 98% and 91%, respectively, during the average follow-up of 46 months in the study [38]. In contrast, a Japanese retrospective study with 31 adult patients with at least 60 months of follow-up showed that approximately 25.8% of patients developed overt nephritis [30]. Large, randomized, prospective, long-term studies are needed to understand if early diagnosis and treatment of SLN can mitigate disease progression, adverse events, and mortality.

## Strengths and limitations of the study

This is the largest retrospective pSLE study of SLN thus far. This study limits interpersonal bias as all the pathology slides were re-reviewed and re-classified by two pathologists based on the latest 2016 ISN/RPS classification for LN. Our study is one of the few studies that reported activity and chronicity indices.

Significant selection bias exists in this study due to the retrospective nature of the study, which might have led to the under-recognition of SLN. Data on 24-h urine collection and microalbuminuria were not available for all patients.

## Conclusion

This retrospective study observed that the odds of having proliferative nephritis were higher in patients with significantly low C3 and C4 levels in this cohort of pSLE patients. The median C3 and C4 levels of both proliferative SLN and OLN were similar and were significantly lower than their non-proliferative counterparts. The non-AA population had higher odds of having proliferative nephritis than the AA population. There was no association between age, sex, presence of anti-ds-DNA Ab or anti-Sm Ab, the decline in eGFR, and proliferative nephritis. Adverse events, including mortality, were noticed in proliferative OLN but not in proliferative SLN during 1-year follow-up. Early diagnosis of treatable SLN might influence outcomes in pSLE patients. Indications with kidney biopsy in pSLE patients without overt kidney findings may require additional scrutiny. Larger, randomized, and long-term studies are needed to investigate the outcomes of SLN patients with normal and low complements.

## Supplementary Information

Below is the link to the electronic supplementary material.

Graphical Abstract (pptx 881 KB)

Supplementary file2 (DOCX 40 KB)

## Data Availability

Raw data were generated at the University of Florida. Derived data supporting the findings of this study are available from the corresponding author, SSM, on request.
