# Supplementary material for: Does kidney biopsy in pediatric lupus patients “complement” the management and outcomes of silent lupus nephritis? Lessons learned from a pediatric cohort
Source: Pediatr Nephrol. 2023 Jan 23;38(8):2669–78. doi: 10.1007/s00467-022-05859-w (PMC10393877; doi:10.1007/s00467-022-05859-w)
Supplement: Supplementary file 1 — Graphical Abstract (pptx 881 KB) [file 467_2022_5859_MOESM1_ESM.pptx]

## Slide 1
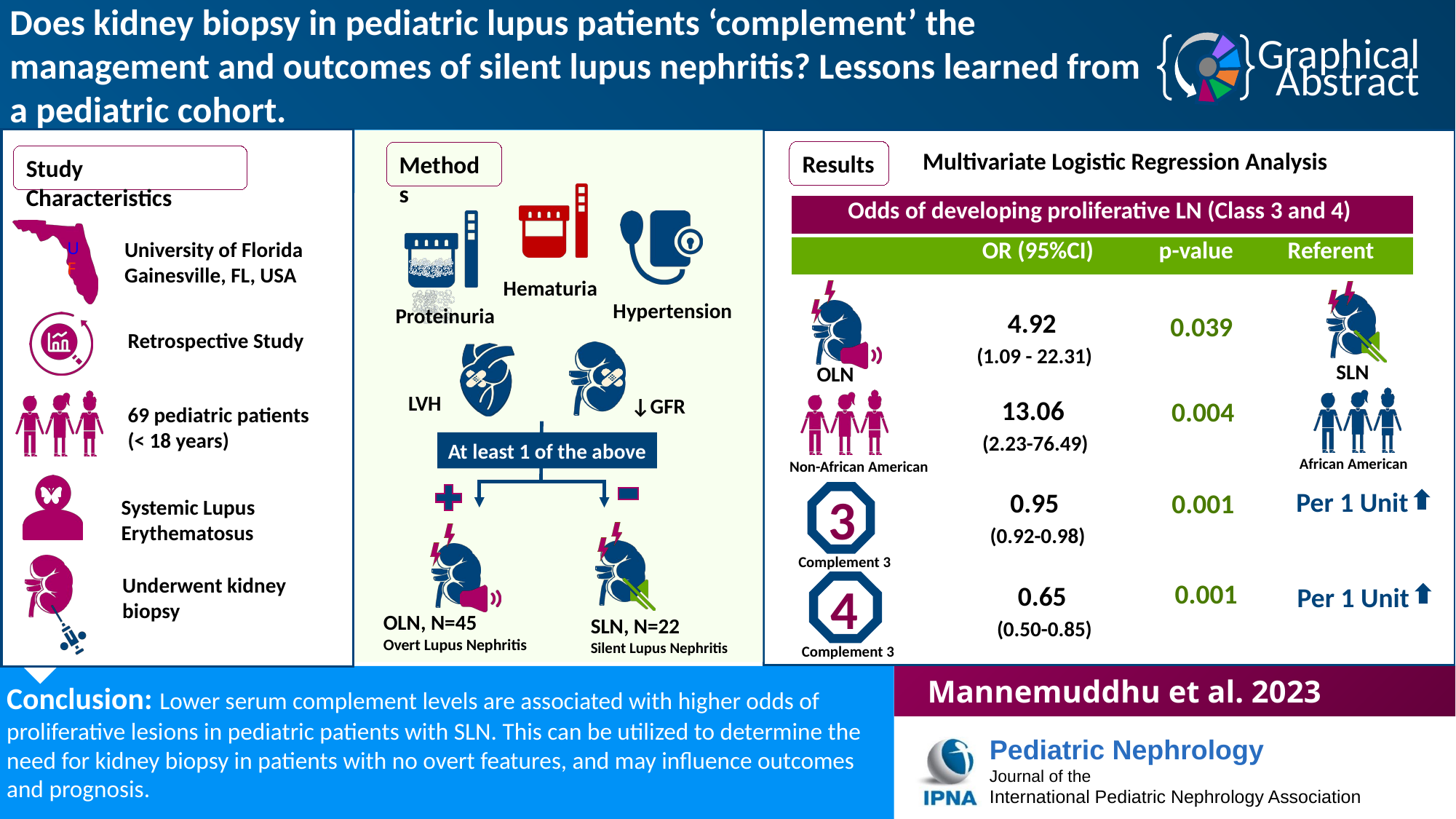

Does kidney biopsy in pediatric lupus patients ‘complement’ the management and outcomes of silent lupus nephritis? Lessons learned from a pediatric cohort.
Study Characteristics
UF
University of Florida
Gainesville, FL, USA
Retrospective Study
69 pediatric patients
(< 18 years)
Systemic Lupus Erythematosus
Underwent kidney biopsy
Methods
Hematuria
Hypertension
Proteinuria
LVH
↓GFR
At least 1 of the above
OLN, N=45
Overt Lupus Nephritis
SLN, N=22
Silent Lupus Nephritis
Multivariate Logistic Regression Analysis
Results
OLN
SLN
4.92
(1.09 - 22.31)
0.039
13.06
(2.23-76.49)
0.004
African American
Non-African American
0.95
(0.92-0.98)
Per 1 Unit
3
0.001
Complement 3
0.65
(0.50-0.85)
4
0.001
Per 1 Unit
Complement 3
| Odds of developing proliferative LN (Class 3 and 4) |
| --- |
| OR (95%CI) p-value Referent |
Mannemuddhu et al. 2023
Conclusion: Lower serum complement levels are associated with higher odds of proliferative lesions in pediatric patients with SLN. This can be utilized to determine the need for kidney biopsy in patients with no overt features, and may influence outcomes and prognosis.
