# Supplementary material for: Does kidney biopsy in pediatric lupus patients “complement” the management and outcomes of silent lupus nephritis? Lessons learned from a pediatric cohort
Source: Pediatr Nephrol. 2023 Jan 23;38(8):2669–78. doi: 10.1007/s00467-022-05859-w (PMC10393877; doi:10.1007/s00467-022-05859-w)
Supplement: Supplementary file 2 — Supplementary file2 (DOCX 40 KB) [file 467_2022_5859_MOESM2_ESM.docx]

**Supplementary Tables:**

**Table S1: ICD-9 and ICD-10 codes used in the retrospective study**

| **ICD 9-10 codes** |  |
| --- | --- |
| M32.14 | Lupus Nephritis |
| M32.9, M32 | SLE |
| Z98.89 | Other specified postprocedural states |
| R94.4 | Abnormal results of kidney function studies |
| R89.9 | Unspecified abnormal findings in specimens from other organs, systems, and tissues |
| I10, I12, I15, I16 | Hypertension, Hypertension and CKD, Secondary hypertension, Hypertensive urgency |
| I11, I11.9, I42 | Hypertensive heart disease, Hypertrophied ventricle |

**Table S1:** ICD-9 and ICD-10 codes used in the retrospective study. SLE: Systemic Lupus Erythematosus; CKD: Chronic Kidney Disease; LVH: Left Ventricular Hypertrophy

**Table S2: Demographics, serological, clinical, and histological findings in proliferative SLN and OLN cohort.**

| **Proliferative (Stage 3&4) SLN vs. OLN** | | | | |
| --- | --- | --- | --- | --- |
| **Factor** |  | **Proliferative**  **SLN** | **Proliferative OLN** | **P- value** |
| **N (%)** |  | 6 (27%) | 27 (57.5%) |  |
| **Female** |  | 5 (83%) | 23 (88%) | 1 |
| **Race** | Asian | 0 | 0 | 0.162 |
|  | African-American | 3 (50%) | 10 (37%) |  |
|  | Hispanic | 1 (17%) | 6 (22%) |  |
|  | Other | 2 (33%) | 2 (7%) |  |
|  | Caucasian | 0 | 8 (30%) |  |
| **Age, mean (SD)** | At diagnosis | 13.6 (5.32) | 13.2(3.32) | 0.775 |
|  | At biopsy | 13.6 (5.32) | 14 (3.65) | 0.853 |
| **dsDNA positive** |  | 6 (100%) | 21 (78%) | 0.562 |
| **Anti-Smith Ab** |  | 4 (66.6%) | 17 (63%) | 0.864 |
| **Anti-RNP Ab** |  | 5 (83%) | 17 (65%) | 0.4 |
| **Anti-SSA Ab** |  | 3 (50%) | 15 (55%) | 0.75 |
| **Anti-SSB Ab** |  | 4 (67%) | 15 (55%) | 0.6 |
| **Anti-Phospholipid Ab** |  | 4 (67%) | 20 (74%) | 0.7 |
| **eGFR (mL/min/1.73m^2^), median (IQR) at biopsy** |  | 123 (121-126) | 110 (67-125) | 0.168 |
| **Hypertension** |  | 0 | 7 (26%) | 0.30 |
| **Random UPCR mg/mg, median (IQR)** |  | 0.16 (0.09-0.18) | 1.22 (0.5-3.3) | 0.007 |
| **Biopsy Results** | Class III | 4 (66.6%) | 8 (30%) |  |
|  | Class IV | 2 (33.3%) | 19 (70%) |  |
| **Activity Index**  **median (IQR)** |  | 5.5 (4-9) | 8 (5-11) | 0.37 |
| **Chronicity Index**  **median (IQR)** |  | 0 | 1 (0-2) | 0.05 |

**Table S2:** Demographics, antibody panel, eGFR, hypertension, urinary findings, and histopathology findings in proliferative SLN vs. OLN patients. eGFR: estimated Glomerular Filtration Rate, IQR: interquartile ranges, UPCR: Urine protein creatinine ratio.

**Table S3: Demographics, clinical and histological findings in proliferative vs. non-proliferative SLN cohort**

| **SLN (Proliferative vs. Nonproliferative)** | | | | |
| --- | --- | --- | --- | --- |
| **Factor** |  | **Proliferative SLN** | **Nonproliferative SLN** | **P- value** |
| **N (%)** |  | 6 (27%) | 16 (73%) |  |
| **Female** |  | 5 (83%) | 13 (81%) | 1 |
| **Race** | Asian | 0 | 0 | 0.84 |
|  | African-American | 3 (50%) | 10 (37.5%) |  |
|  | Hispanic | 1 (17%) | 0 |  |
|  | Other | 2 (33%) | 2 (12.5%) |  |
|  | Caucasian | 0 | 1 (6%) |  |
| **Age, mean (SD)** | At diagnosis | 13.6 (5.32) | 12.3 (4.32) | 0.54 |
|  | At biopsy | 13.6 (5.32) | 12.7 (4.50) | 0.69 |
| **Complement**  **(mg/dL), median (IQR)** | C3 | 32.5 (24-34) | 63 (46.5-88.5) | **0.007** |
|  | C4 | 2.5 (2-4) | 8 (4-9.5) | **0.02** |
| **dsDNA positive** |  | 6 (100%) | 12 (67%) | 0.54 |
| **Anti-Smith Ab** |  | 4 (66.6%) | 31 (81%) | 0.58 |
| **Anti-RNP Ab** |  | 5 (83%) | 15 (94%) | 0.48 |
| **Anti-SSA Ab** |  | 3 (50%) | 8 (53%) | 1 |
| **Anti-SSB Ab** |  | 2 (33%) | 4 (27%) | 1 |
| **Anti-Phospholipid Ab** |  | 2 (33%) | 3 (21%) | 0.6 |
| **eGFR (mL/min/1.73m^2^), median (IQR) at biopsy** |  | 123 (121-126) | 130.5 (115-140.8) | 0.46 |
| **Hypertension** |  | 0 | 0 |  |
| **Random UPCR mg/mg, median (IQR)** |  | 0.16 (0.09-0.18) | 0.11 (0.06-0.14) | 0.3 |
| **Biopsy Results** | Class I | - | 3 (19%) |  |
|  | Class II | - | 11 (69%) |  |
|  | Class III | 4 (67%) | - |  |
|  | Class IV | 2 (33%) | - |  |
|  | Class V | - | 2 (12%) |  |
|  | Class VI | - | 0 |  |

**Table S3:** Demographics, lab results, eGFR, hypertension, urinary finding, and kidney biopsy findings in proliferative vs. non-proliferative SLN patients.

**Table S4: Demographics, clinical, and histological findings in proliferative vs. non-proliferative OLN cohorts.**

| **OLN (Proliferative vs. Nonproliferative)** | | | | |
| --- | --- | --- | --- | --- |
| **Factor** |  | **Proliferative OLN** | **Nonproliferative OLN** | **P- value** |
| **N (%)** |  | 27 (57.5%) | 20 (42.5%) |  |
| **Female** |  | 23 (88%) | 16 (80%) |  |
| **Race** | Asian | 0 | 1 (5%) | 0.3 |
|  | African-American | 10 (37%) | 15 (75%) |  |
|  | Hispanic | 6 (22%) | 2 (10%) |  |
|  | Other | 2 (7%) | 1 (5%) |  |
|  | Caucasian | 8 (30%) | 1 (5%) |  |
| **Age, mean (SD)** | At diagnosis | 13.2(3.32) | 14.05 (3.02) | 0.36 |
|  | At biopsy | 14 (3.65) | 14.5 (3.22) | 0.6 |
| **Complement**  **(mg/dL), median (IQR)** | C3 | 44 (27-54) | 72 (52-88) | **0.002** |
|  | C4 | 4 (3-8) | 9.5 (7-16.5) | **0.0001** |
| **dsDNA positive** |  | 21 (78%) | 15 (75%) | 1 |
| **Anti-Smith Ab** |  | 17 (63%) | 16 (80%) | 0.11 |
| **Anti-RNP Ab** |  | 17 (63%) | 16 (80%) | 0.52 |
| **Anti-SSA Ab** |  | 15 (55%) | 9 (45%) | 0.56 |
| **Anti-SSB Ab** |  | 12 (44%) | 7 (35%) | 0.55 |
| **Anti-Phospholipid Ab** |  | 7 (26%) | 3 (15%) | 0.48 |
| **eGFR (mL/min/1.73m^2^), median (IQR) at biopsy** |  | 110 (67-125) | 116 (98-149) | 0.23 |
| **Hypertension** |  | 7 (26%) | 2 (10%) | 0.27 |
| **Random UPCR mg/mg, median (IQR)** |  | 1.22 (0.5-3.3) | 0.49 (0.14-3.5) | 0.2 |
| **Biopsy Results** | Class I | - | 1 (5%) |  |
|  | Class II | - | 10 (50%) |  |
|  | Class III | 8 (30%) | - |  |
|  | Class IV | 19 (70%) | - |  |
|  | Class V | - | 9 (45%) |  |
|  | Class VI | - | 0 |  |

**Table S4:** Demographics, lab results, eGFR, hypertension, urinary finding, and kidney biopsy findings in proliferative vs. non-proliferative OLN patients.

**Table S5: SLN vs. OLN- Clinical features and hematology findings**

| **Clinical Features/ Hematology** | **Silent LN** | **Overt LN** | **P-value** |
| --- | --- | --- | --- |
| **Malar Rash** | 11 (50%) | 22 (47%) | 0.08 |
| **Mucosal Ulcers** | 6 (27%) | 10 (21%) | 0.58 |
| **Arthritis** | 15 (68%) | 30 (64%) | 0.72 |
| **Serositis** | 7 (32%) | 16 (34%) | 0.85 |
| **Myositis** | 4 (18%) | 6 (13%) | 0.71 |
| **Fever** | 11 (50%) | 26 (55%) | 0.56 |
| **Headache** | 9 (41%) | 14 (30%) | 0.36 |
| **Psychosis** | 1 (5%) | 1 (2%) | 0.53 |
| **Anemia** | 18 (82%) | 33 (70%) | 0.31 |
| **Leukopenia** | 17 (77%) | 27 (57%) | 0.11 |
| **Thrombocytopenia** | 5 (23%) | 14 (30%) | 0.54 |

**Table S5:** Clinical features, and hematology findings in SLN vs OLN

**Table S6: Proliferative SLN vs. OLN- Clinical features and hematology findings**

| **Clinical Features / Hematology** | **Proliferative SLN** | **Proliferative OLN** | **P-value** |
| --- | --- | --- | --- |
| **Malar Rash** | 3 (50%) | 14 (52%) | 1 |
| **Mucosal Ulcers** | 3 (50%) | 7 (26%) | 0.34 |
| **Arthritis** | 6 (100%) | 19 (70%) | 0.30 |
| **Serositis** | 3 (50%) | 9 (33%) | 0.64 |
| **Myositis** | 1 (17%) | 3 (11%) | 1 |
| **Fever** | 4 (67%) | 17 (63%) | 1 |
| **Headache** | 2 (33%) | 11 (41%) | 1 |
| **Psychosis** | 0 | 0 | - |
| **Anemia** | 6 (100%) | 19 (70%) | 0.30 |
| **Leukopenia** | 5 (83%) | 16 (60%) | 0.37 |
| **Thrombocytopenia** | 1 (17%) | 8 (30%) | 1 |

**Table S6:** Clinical features, and hematology findings in proliferative SLN vs. OLN sub-groups
